# Genetically enhancing the expression of chemokine domain of CX_3_CL1 fails to prevent tau pathology in mouse models of tauopathy

**DOI:** 10.1186/s12974-018-1310-6

**Published:** 2018-09-25

**Authors:** Shane M. Bemiller, Nicole M. Maphis, Shane V. Formica, Gina N. Wilson, Crystal M. Miller, Guixiang Xu, Olga N. Kokiko-Cochran, Ki-Wook Kim, Steffen Jung, Judy L. Cannon, Samuel D. Crish, Astrid E. Cardona, Bruce T. Lamb, Kiran Bhaskar

**Affiliations:** 1 Department of Molecular Genetics and Microbiology, University of New Mexico, Albuquerque, NM 87113 USA; 2 Department of Neurology, University of New Mexico, MSC08 4660, 1 University of New Mexico, Albuquerque, NM 87131 USA; 3 Department of Neurosciences, Cleveland Clinic Foundation, Cleveland, OH 44195 USA; 4 Department of Immunology, Weizmann Institute of Science, 76100 Rehovot, Israel; 5 Kent State University, Kent, OH 44242 USA; 6 Stark Neurosciences Research Institute, Indiana University School of Medicine, Indianapolis, IN 46202 USA; 7 Department of Pharmacology, Northeast Ohio Medical School, Rootstown, OH 44272 USA; 8 Department of Biology, University of Texas San Antonio, West Campus/Tobin lab MBT 1.216, San Antonio, TX 78249 USA; 9 Department of Neuroscience, College of Medicine, The Ohio State University, Columbus, OH 43210 USA; 10 Department of Pathology and Immunology, Washington University School of Medicine in St. Louis, 660 S. Euclid Ave., Campus Box 8118, St. Louis, MO 63110 USA

**Keywords:** Alzheimer’s disease, Tauopathies, Tau, Microglia, Neuroinflammation, CX_3_CR1, CX_3_CL1

## Abstract

**Background:**

Fractalkine (CX_3_CL1) and its receptor (CX_3_CR1) play an important role in regulating microglial function. We have previously shown that *Cx*_*3*_*cr1* deficiency exacerbated tau pathology and led to cognitive impairment. However, it is still unclear if the chemokine domain of the ligand CX_3_CL1 is essential in regulating neuronal tau pathology.

**Methods:**

We used transgenic mice lacking endogenous *Cx*_*3*_*cl*1 (*Cx*_*3*_*cl1*^−/−^) and expressing only obligatory soluble form (with only chemokine domain) and lacking the mucin stalk of CX_3_CL1 (referred to as *Cx*_*3*_*cl1*^105Δ^ mice) to assess tau pathology and behavioral function in both lipopolysaccharide (LPS) and genetic (hTau) mouse models of tauopathy.

**Results:**

First, increased basal tau levels accompanied microglial activation in *Cx*_*3*_*cl1*^105Δ^ mice compared to control groups. Second, increased CD45^+^ and F4/80^+^ neuroinflammation and tau phosphorylation were observed in LPS, hTau/*Cx*_*3*_*cl1*^−/−^, and hTau/*Cx*_*3*_*cl1*^105Δ^ mouse models of tau pathology, which correlated with impaired spatial learning. Finally, microglial cell surface expression of CX_3_CR1 was reduced in *Cx*_*3*_*cl1*^105Δ^ mice, suggesting enhanced fractalkine receptor internalization (mimicking *Cx*_*3*_*cr1* deletion), which likely contributes to the elevated tau pathology.

**Conclusions:**

Collectively, our data suggest that overexpression of only chemokine domain of CX_3_CL1 does not protect against tau pathology.

**Electronic supplementary material:**

The online version of this article (10.1186/s12974-018-1310-6) contains supplementary material, which is available to authorized users.

## Background

Fractalkine signaling in the CNS represents a unique microglial-neuron receptor-ligand pair, where fractalkine (CX_3_CL1) is expressed by neurons and its cognate receptor CX_3_CR1 is exclusively expressed by the CNS resident microglia [1]. CX_3_CL1 is a 373-amino acid protein, which contains an extracellular chemokine domain linked to a mucin-like stalk [2, 3]. CX_3_CL1 is functional in its membrane-bound form but can also be cleaved through metalloprotease (ADAM10/ADAM17) activity to produce a ~ 95-kDa soluble moiety [4, 5]. It has been proposed that the heavily glycosylated mucin-like stalk of fractalkine provides rigidity to the chemokine domain for the adhesive potency of the chemokine domain during patrolling/crawling behavior [6]. Several mouse models have been used to elucidate the role of fractalkine in mediating neurodegenerative and neuroinflammatory processes [7–11].

CX_3_CL1-CX_3_CR1 signaling is regulated through direct neuron-microglia interaction, which acts to tether microglia until pathological activation, via an inflammatory influence, or through normal physiological activity, which disrupts this interaction through the cleavage of CX_3_CL1 [12, 13]. Disruption of CX_3_CL1-CX_3_CR1 signaling by chemical or genetic manipulation induces dramatic morphological activation and altered levels of scavenger/inflammatory receptors on the cell surface, alterations in pro-inflammatory chemokine production, and over-sensitization to pathological insults [14–17].

Previous studies from our group have explored the role of CX_3_CL1 signaling in the context of Alzheimer’s disease (AD) and related dementias. Notably, we found that disrupting the CX_3_CL1-CX_3_CR1 signaling axis reduces Aβ burden with concomitant increases in pro-inflammatory IL-1 and heightened microglial activation in both APPPS1/*Cx*_*3*_*cr1*^−/−^ and APPPS1/*Cx*_*3*_*cl1*^−/−^ transgenic mouse models of AD [18]. Interestingly, this phenomenon was unaffected by the presence of soluble CX_3_CL1 [18]. In a separate study, converse to the protective anti-amyloid phenotype observed in APPPS1/*Cx*_*3*_*cr1*^−/−^ mice, deletion of *Cx*_*3*_*cr1* in hTau mice resulted in hyperphosphorylation and aggregation of tau, worsened cognitive function, and increased microglial inflammation [17]. This effect was regulated via the same IL-1-p38 MAPK axis [17, 19]. The dichotomy between the two studies likely stems from the type of pathological insults present, namely Aβ is extracellular whereas hyperphosphorylated tau exists primarily intraneuronally [20]. The precise mechanism of how disrupting the CX_3_CL1-CX_3_CR1 signaling affects the microglia either to a beneficial (in the case of Aβ study) or to a detrimental degree (in the hTau study) is still unclear. However, it is possible that the IL-1β promotes phagocytic phenotype of microglia in clearing Aβ (in case of APPPS1/*Cx*_*3*_*cr1*^−/−^ and APPPS1/*Cx*_*3*_*cl1*^−/−^ mice), while causing collateral damage (for example, over-activation of p38 MAPK) in neurons and leading to tau hyperphosphorylation [17–19]. Seemingly, contrary work demonstrated that *Cx*_*3*_*cl1* overexpression through viral transfection models reduces tau and α-synuclein pathology [10, 21]. The present study seeks to determine if genetically expressing only the soluble chemokine domain of CX_3_CL1 could prevent tau pathology in both chemical (LPS) and genetic (hTau) mouse models of tauopathy.

## Methods

### Experimental animals

A mouse line (*Cx*_*3*_*cl1*^105Δ^) exclusively expressing obligate soluble CX_3_CL1 featuring only the chemokine domain, without the mucin stalk, was generated by introducing bacterial artificial chromosome (BAC) transgene encoding truncated CX_3_CL1 (*B6.Cg-Tg(Cx*_*3*_cl1*)1Jung/J RRID:IMSR_JAX:027119) to *Cx*_*3*_*cl1*^*−/−*^ mice (RRID: MGI_2388041) [22]. For the current study, hTau^+/−^;*Mapt*^−/−^ [23] (acquired from the Jackson Laboratory) which expressed all six isoforms of the human MAPT under the control of the endogenous human *MAPT* promoter and backcrossed into *Cx*_*3*_*cl1*^105Δ^ animals [22] was subsequently intercrossed to generate both hTau^+/−^;*Mapt*^−/−^/*Cx*_*3*_*cl1*^−/−^ (referred as “hTau/*Cx*_*3*_*cl1*^−/−^”) and hTau^+/−^;*Mapt*^−/−^/*Cx*_*3*_*cl1*^−/−^/ *Cx*_*3*_*cl1*^105Δ^ (referred as “hTau/*Cx*_*3*_*cl1*^105Δ^”). Mice were housed in both the Cleveland Clinic Biological Resources Unit and University of New Mexico Animal Research Facility. Both facilities are fully accredited by the Association and Accreditation of Laboratory Animal Care. The Institutional Animal Care and Use Committee at respective institutions approved all experimental procedures.

### Lipopolysaccharide injections

Three milligrams per kilogram b.w. LPS (Sigma-Aldrich) was administered intraperitoneally (i.p) to 2-month-old mice and sacrificed 24 h post-injection. The hemi-brains were post-fixed in 4% paraformaldehyde (PFA) followed by cryopreservation in 30% sucrose for immunohistochemistry (IHC) experiments. The remaining half of the brains were microdissected into the hippocampal and cortical fractions and snap frozen in liquid nitrogen and stored at − 80 °C for biochemical analysis.

### Western blotting

**Fig. 1:**
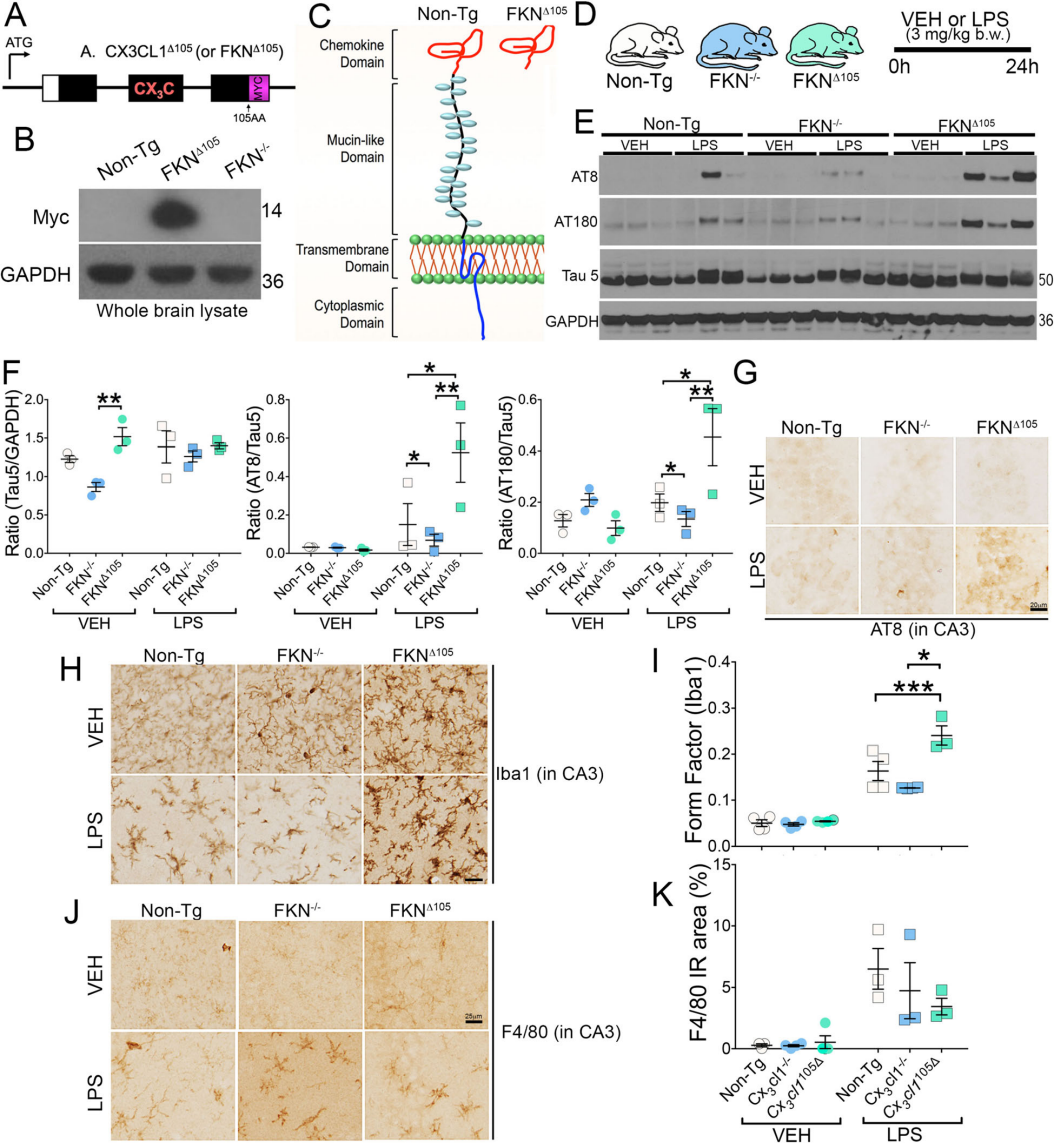
LPS-induced tau phosphorylation and microglial activation are exacerbated in *Cx*_*3*_*cl1*^105Δ^ mice. **a**–**d** Two-month-old fractalkine (*Cx*_*3*_*cl1*^−/−^)-deficient mice and the mice exclusively expressing the chemokine domain (lacking the mucin-like domain, red) (CX_3_CL1^105Δ^) with a Myc tag were injected with LPS (3 mg/kg b.w; i.p) or vehicle (VEH, Hank’s balanced salt solution or HBSS) and sacrificed 24 h post-injection. **e**–**f** Western blotting of the hippocampi revealed significantly increased total tau (Tau5) (> 1.5-fold) in VEH-treated *Cx*_*3*_*cl1*^105Δ^ vs. *Cx*_*3*_*cl1*^−/−^ mice (mean + SEM; **p < 0.01; *n* = 3; two-way ANOVA followed by Tukey’s post hoc test). Both AT8/Tau5 and AT180/Tau5 ratios were significantly higher in LPS-treated *Cx*_*3*_*cl1*^105Δ^ compared to LPS-treated *Cx*_*3*_*cl1*^−/−^ or Non-Tg mice (mean + SEM; *p < 0.05; **p < 0.01; *n* = 3; two-way ANOVA with Tukey’s post hoc test). **g** Immunohistochemistry (IHC) analysis revealing a modest increase in AT8 (pS199/pS202 tau) among experimental genotypes or between VEH- or LPS-injected mice in the CA3 hippocampal areas. Scale bar, 20 μm. **h**–**k** IHC showing elevated Iba1^+^/F4/80^+^ reactive microglia in VEH-treated *Cx*_*3*_*cl1*^105Δ^ mice that is enhanced with LPS treatment. Quantification reveals statistically higher form factor units (higher number means more towards circular contour) for Iba1^+^ microglia in Non-Tg and *Cx*_*3*_*cl1*^105Δ^ mice in LPS-treated groups (mean + SEM; ***p < 0.0001 vs. **p < 0.01 for Non-Tg with LPS; two-way ANOVA with Tukey’s post hoc test; *n* = 3–6 mice per group). Scale bars (**h**, **j**) 25 μm

**Fig. 2:**
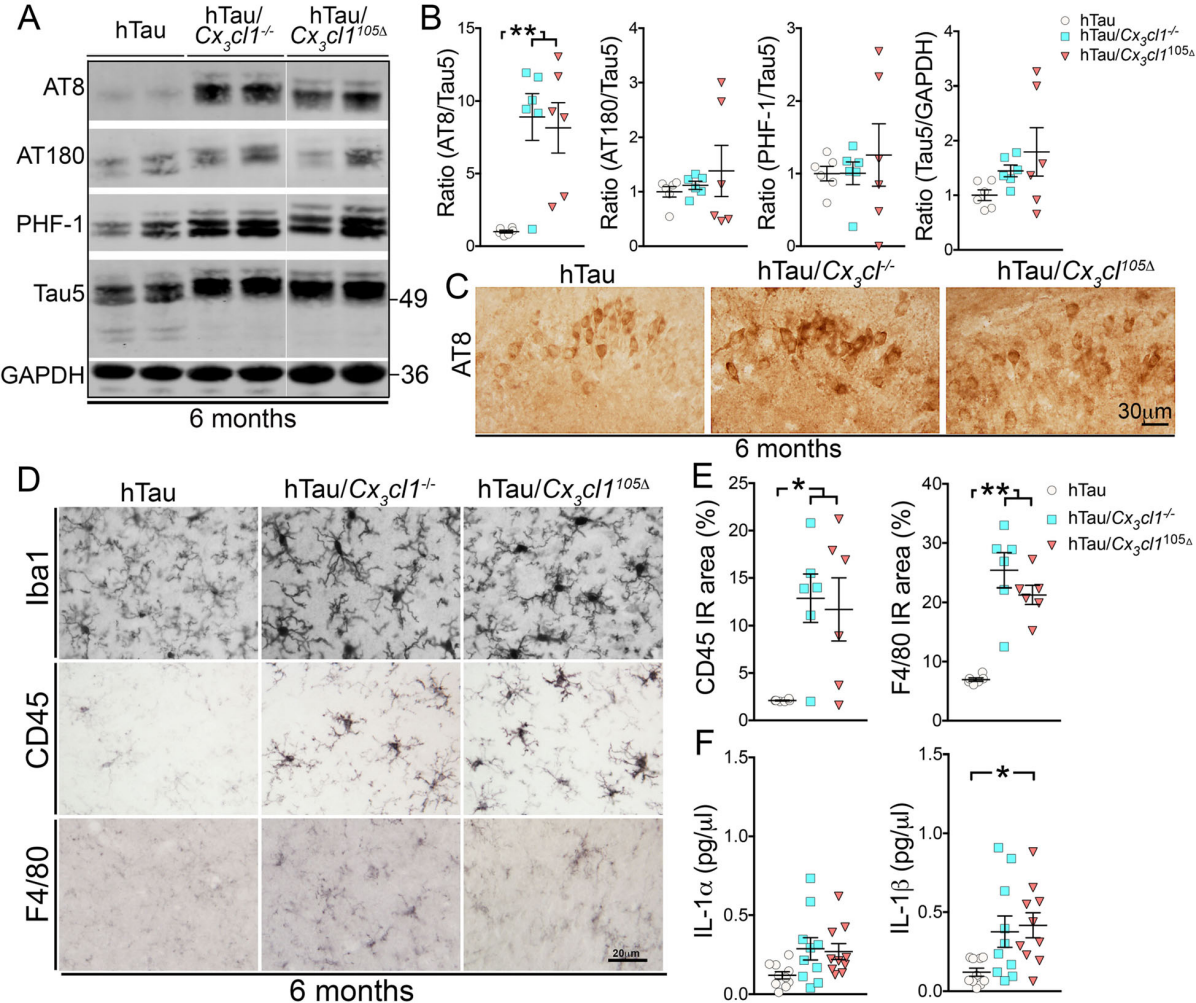
Increased tau pathology, IL-1α/IL-1β, and microglial activation in 6-month-old hTau/*Cx*_*3*_*cl1*^−/−^ and hTau/*Cx*_*3*_*cl1*^105Δ^ mice. **a**, **b** Western blotting revealed increases in AT8^+^ tau in the hippocampus of hTau/*Cx*_*3*_*cl1*^−/−^ and hTau/*Cx*_*3*_*cl1*^105Δ^ mice compared to hTau controls. **c** AT8 IHC revealed increased reactivity in the CA3 regions of hTau/*Cx*_*3*_*cl1*^−/−^ and hTau/*Cx*_*3*_*cl1*^105Δ^ groups compared to hTau controls. Scale bar, 30 μm. **d** Significant increases in both CD45 and F4/80 immunoreactivities were detected and quantified (**e**) in the cortex of hTau/*Cx*_*3*_*cl1*^−/−^ and hTau/*Cx*_*3*_*cl1*^105Δ^ mice compared to controls. **f** A significant increase in IL-1α and IL-1β was observed in both hTau/*Cx*_*3*_*cl1*^−/−^ and hTau/*Cx*_*3*_*cl1*^105Δ^ mice via ELISA. *n* = 6 mice per group except for ELISA (*n* = 10). Three independent experiments were performed for each analysis. Error bars represent SEM. One-way ANOVA followed by Tukey’s post hoc test: *p < 0.05, **p < 0.01, ***p < 0.001

Microdissected cortical and hippocampal fractions were homogenized using T-PER reagent (Thermo #78510) containing phosphatase and protease inhibitor cocktails (Thermo #78429, #78443; Sigma-Aldrich #p5726) and briefly sonicated at 20% amplitude for 10 s. Homogenates were centrifuged at 15,000 rpm, and the protein in the supernatant measured via BCA assay (Thermo #23225). Total protein (30–60 μg) were resolved by SDS-PAGE, transferred to the PVDF membranes (#IPFL10100 Millipore), and probed with phosphorylated tau antibodies (AT8 for pS199/pS202/pT205, AT180 for pT231 at 1:5000; Thermo; and PHF-1; 1:10,000; a generous gift from Dr. Peter Davies), total tau (Tau5 1:10,000; Thermo), and GAPDH (1:20,000; Millipore) (loading control). The membranes were incubated with near-IR conjugated (Thermo #A11371, #A11367) or HRP-conjugated secondary antibodies (Jackson ImmunoResearch), visualized, and either quantitated using LICOR Odyssey imaging systems (for the data presented in Fig. 2) or developed with enhanced chemiluminescence reagent and quantified by Alpha Innotech® software (for the data presented in Fig. 1).

### Immunohistochemistry

Sagittal free-floating 30-μm sections were subjected to standard sodium citrate antigen retrieval for 10 min at 95 °C followed by blocking in normal goat serum containing 0.1% Triton-X. The sections were incubated overnight with AT8, AT180, or myeloid cell surface markers (CD45 #MCA1388 1:500; BioRad; F4/80 #MCA497G 1:500 BioRad; Iba1 #019-19741 1:500; Waco) and respective biotinylated secondary antibodies (Vector Laboratories Catalog # BA-2000, BA-9400, BA-1000) and developed using 3-3′-diaminobenzidine with or without the nickel enhancer. Data were quantified using percentage immunoreactive area or form factor analysis [24, 25]. First, percent immunoreactive area for CD45 and F4/80 was processed using ImageJ, where five random fields per section were manually defined as region of interest (ROI) in three random sections (focusing only on the ones containing dorsal hippocampal region) per mouse and in *n* = 6 mice per genotype and consistently scored to detect percentage of CD45 and F4/80 immunoreactive area using ImageJ software. Briefly, first, the RGB images were converted into 8-bit gray scale, and then the images were processed to adjust the threshold, which was kept constant for all images. Finally, using the analyze tool in ImageJ, the total immunoreactive area per field was scored by an automated routine. After completing the scoring for all sections, the mean percentage area along with standard error of mean was plotted. For quantifying the roundness of Iba1+ microglia, we utilized *form factor* (FF) algorithm in the ImagePro Plus® software. FF measures the roundness of an object; in this case, it was Iba1+ microglia. We [25] and others [24] have previously described FF-based quantification of microglial contour as an indirect measure of its phagocytic/morphological activation state. Briefly, three images at random were taken in three different frontal cortical sections from each animal with at least three animals per group. FF measures the contour irregularity of a cell, i.e., FF is higher (approaches 1.0) in bushy cells, characterized by larger cell bodies that are less ramified, while morphologically “resting” microglia appear smaller cell bodies with abundant branches of regularly ramified processes, which would have lower FF values (closer to 0.0). A total of 235 (Non-Tg-Veh), 184 (Non-Tg-LPS), 220 (*Cx*_*3*_*cl1*^*−/−*^-Veh), 192 (*Cx*_*3*_*cl1*^*−/−*^-LPS), 234 (*Cx*_*3*_*cl1*^*105Δ*^-Veh), and 200 (*Cx*_*3*_*cl1*^*105Δ*^-LPS) microglial cells were scored for FF analysis.

### Behavioral analysis

#### Morris water maze

Mice underwent a 3-day training using a visible platform, which was relocated to different quadrants of an opaque, water-filled maze each of the four trials per day. The first 3 days of *visible platform training* was to allow animals to learn the procedures of the task (i.e., swim and get onto the platform to escape from the pool). Next, the animals received 5 days of *memory testing* in which the platform was submerged and remained in a constant location with static spatial cues around the room. Latency to reach a stationary hidden platform was recorded across four separate trials per day, for all 5 days. Mice were allotted 60 s to reach the platform during both training and experimental days. Latency to reach the platform, swim speed, within-day learning, and across-day learning was examined to determine cognitive differences between experimental genotypes. We analyzed three key parameters relevant to learning trends: (a) *acquisition index* is a measure comparing learning within each trial day across four trials; (b) *savings index* is a measure of memory consolidation from the final trial of one testing day to the first trial of the following day; and (c) *slope* plots the trajectory of learning curve. These indices were scored for all 5 days as previously described [26].

#### Y-maze

The Y-maze is used to assess spatial working memory during a 5-min trial where each mouse is allowed to freely explore each arm of the Y-maze [27]. Total arm entries, repeat ratio (defined as the number of times a mouse enters the same arm twice over a total number of arm entries), and the spontaneous alternation (defined as when a mouse consecutively entered three different arms) were recorded, as previously described [17].

#### Multiplex ELISA assay

Cytokine levels were all normalized to total protein concentration following BCA assay. Multiplex assays were performed according to the manufacturer’s instructions using reagents provided with the kit (Invitrogen Mouse 20-plex Cytokine Panel, Cat# LMC0006M). Following sample incubation, plates were washed, incubated with streptavidin-RPE for 30 min, washed three times, and followed by a final addition of 125 μl wash solution to all wells. The plates were read on a Luminex Magpix unit (Life Technologies), and initial analyses were performed by Xponent software and results exported into Microsoft Excel for further processing. The sample size was set to 50 μl, and the minimum count was set to 100 events/bead regions.

#### Flow cytometry

Mononuclear cells were isolated via a density centrifugation technique at the interface of a 30/70% Percoll gradient (Fisher Scientific #17-5445-01), as previously described [28]. Cells were blocked using Fc blocking reagent (BD Biosciences #553141) for 10 min before a 30-min incubation with fluorophore-conjugated flow cytometry antibodies against CD11b (FITC; BD Biosciences #553310), CD45 (APC; BD Biosciences; #559864), and CX3CR1 (PE; BD Biosciences #565798). Data were acquired using BD Biosciences Fortessa Flow Cytometer and analyzed using FlowJo single cell analysis software. Fifty thousand events were minimally collected before data processing. Mean fluorescent intensity was utilized in conjunction with total event counts in order to quantify the number of brain-resident microglia (CD11b^+^/CD45^low^ cells) and relative expression of CX3CR1.

### Statistical analysis

Data are presented as mean ± SEM unless otherwise noted. Comparisons between two groups were analyzed using Student’s *t* test (two-tailed; unpaired) at 95% confidence interval. Multiple group comparison or multiple comparisons were analyzed using ANOVA or MANOVA followed by Tukey’s or Dunnett’s post hoc tests. The analysis was performed using Prism GraphPad or SPSS software. Significance was determined at *p < 0.05, **p < 0.01, and ***p < 0.001. Individuals who were blinded to the genotype/treatment groups performed the data analysis.

## Results

### Enhanced microglial activation in *Cx*_*3*_*cl1*^105Δ^ mice during LPS-induced endotoxemia

To explore the effect of neuronal *Cx*_*3*_*cl1* deficiency and the overexpression of the shed fractalkine moiety on LPS-induced tau pathology, we utilized fractalkine-deficient (endogenous *Cx3cl1*^*−/−*^) and cleaved soluble fractalkine (*Cx*_*3*_*cl1*^105Δ^)-expressing transgenic mice, which express only the soluble chemokine domain of CX_3_CL1 [22]. Previously, this model revealed a differential requirement for soluble and membrane-bound CX_3_CL1 in the context of dendritic macrophage processes within the gut epithelium [22]. First, we confirmed the expression of *Cx*_*3*_*cl1*^105Δ^ in the whole brain lysate via detection of a c-Myc tag present in the C-terminal end of the *Cx*_*3*_*cl1*^105Δ^ BAC construct (Fig. 1a-c) [22]. Interestingly, the mRNA levels of *Cx3cl1* were significantly higher in *Cx*_*3*_*cl1*^105Δ^ mice compared to non-transgenic controls (Additional file 1: Figure S1A). However, the protein levels of soluble CX_3_CL1 were comparable to that of non-transgenic mice (Additional file 1: Figure S1B).

Previous work from our lab has demonstrated that LPS induces tauopathy as early as 24 h following administration [17]. We administered LPS (3 mg/kg b.w., single dose; i.p) to 2-month non-transgenic C57BL/6J (Non-Tg), *Cx*_*3*_*cl1*^−/−^, and *Cx*_*3*_*cl1*^105Δ^ mice (Fig. 1d). Based on our previous reports that LPS leads to tau phosphorylation within 24 h [17, 29], mice were sacrificed 24 h post-injection to determine the alterations in tau phosphorylation and microglial activation. Western blotting revealed a significant (> 1.5-fold) increase in the total tau (Tau5) levels in vehicle-treated *Cx*_*3*_*cl1*^105Δ^ mice compared to *Cx*_*3*_*cl1*^−/−^ mice (Fig. 1e, f). No differences were detected in the basal level of tau phosphorylation among LPS-treated Non-Tg, *Cx*_*3*_*cl1*^−/−^, and *Cx*_*3*_*cl1*^105Δ^ groups. Notably, LPS administration elevated phosphorylated tau levels at AT8 (S199/S202/T205) and AT180 (T231) sites in Non-Tg and *Cx*_*3*_*cl1*^105Δ^ mice that were nearly two- to fourfold elevated compared to vehicle-treated groups, whereas *Cx*_*3*_*cl1*^−/−^ mice revealed only a modest increase in AT8^+^ tau in LPS-injected groups (Fig. 1e, f). However, the LPS-induced AT8 positivity was more robust in 6-month-old *Cx*_*3*_*cl1*^−/−^ mice (data not shown).

Immunohistochemical analysis revealed a moderate increase in AT8 immunoreactivity in the hippocampus (CA3) of *Cx*_*3*_*cl1*^105Δ^ mice compared with other genotypes (Fig. 1g). Furthermore, Iba1+ microglial immunostaining with subsequent form factor quantitative analysis revealed an increase in microglial activation with LPS in all three genotypes tested (Fig. 1h, i). Reactive microglia displayed thick, less ramified processes in Non-Tg, *Cx*_*3*_*cl1*^−/−^, and *Cx*_*3*_*cl1*^105Δ^ mice treated with LPS compared with their respective vehicle-injected controls (Fig. 1h). While the expression of a major macrophage marker—F4/80—appeared elevated with LPS treatment in the IHC images, the differences were not statistically significant due to large variability. We also did not detect any differences among experimental genotypes in either vehicle or LPS-injected groups (Fig. 1j-k).

### Overexpression of only chemokine domain of CX_3_CL1 fails to mitigate microglial activation and tau pathology induced by *Cx*_*3*_*cl1* deficiency in hTau mice

To determine the effect of *Cx*_*3*_*cl1* deficiency and specific effects of *Cx*_*3*_*cl1*^105Δ^ expression in hTau mice, the brains of 6-month-old hTau, hTau/*Cx*_*3*_*cl1*^−/−^, and hTau/*Cx*_*3*_*cl1*^105Δ^ mice were analyzed. Significant increases in AT8 site tau phosphorylation, but not AT180, PHF-1 sites, or total tau (Tau5) were detected in the hippocampus of hTau/*Cx*_*3*_*cl1*^−/−^ and hTau/*Cx*_*3*_*cl1*^105Δ^ mice compared to hTau mice (Fig. 2a, b). Immunohistochemical analysis revealed increased AT8 immunoreactivity in hTau/*Cx*_*3*_*cl1*^−/−^ and hTau/*Cx*_*3*_*cl1*^105Δ^ mice compared to hTau mice in the CA3 region of the hippocampus, where the AT8+ tau pathology was robust (Fig. 2c). Significant increases in Iba1, CD45, and F4/80 immunoreactivities were detected in hTau/*Cx*_*3*_*cl1*^−/−^ and hTau/*Cx*_*3*_*cl1*^105Δ^ mice compared to hTau mice (Fig. 2d, e). Multiplex ELISA analysis of hippocampal lysates revealed a significant increase in inflammatory IL-1α in hTau/*Cx*_*3*_*cl1*^−/−^ mice and a modest increase (*p* = 0.07) for hTau/*Cx*_*3*_*cl1*^105Δ^ mice compared to hTau mice (Fig. 2f). Notably, IL-1β levels were significantly elevated in both hTau/*Cx*_*3*_*cl1*^−/−^ and hTau/*Cx*_*3*_*cl1*^105Δ^ mice compared to hTau mice (Fig. 2f), which is consistent with our previous studies linking increased IL-1β production to the microglial p38 MAPK signaling pathway [17–19].

### Microglial cell surface level of CX_3_CR1 is significantly reduced in *Cx*_*3*_*cl1*^105Δ^ mice, mimicking a *Cx3cr1* deficiency phenotype

**Fig. 3:**
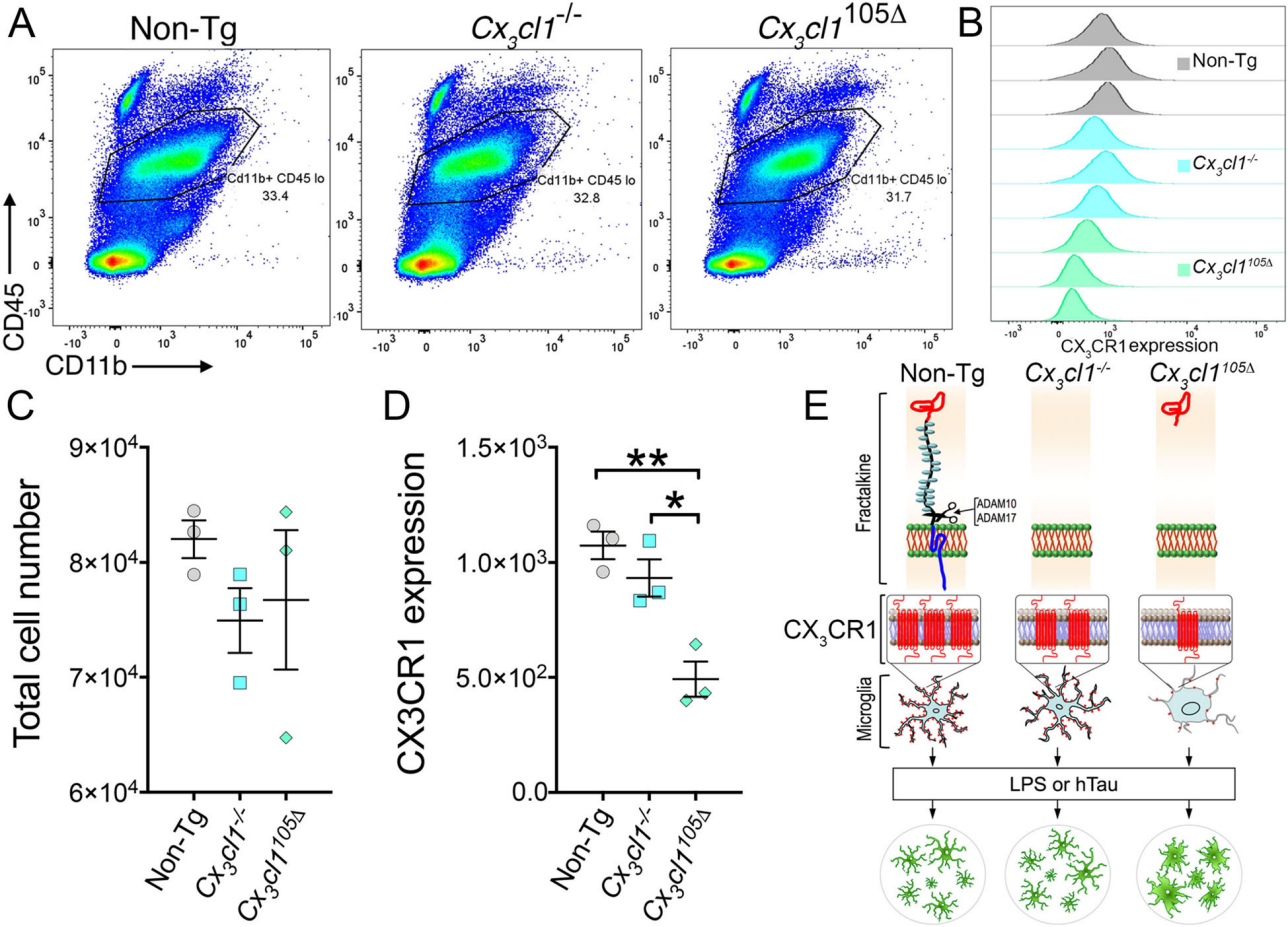
Expression of the microglial CX_3_CR1 is decreased in *Cx*_*3*_*cl1*^105Δ^ mice. **a**, **c** Flow cytometry on isolated brain mononuclear cells revealed no alteration in the total number of resident microglia in Non-Tg, *Cx*_*3*_*cl1*^−/−^, or *Cx*_*3*_*cl1*^105Δ^ mice (Cd11b^+^/CD45^low^). **b**, **d** Overall decreased CX_3_CR1 expression in the CD11b^+^CD45^low^ (microglial population) in *Cx*_*3*_*cl1*^105Δ^ mice compared to Non-Tg and *Cx*_*3*_*cl1*^−/−^ mice (mean + SEM; one-way ANOVA followed by Tukey’s post hoc test: *p < 0.05, **p < 0.01; *n* = 3 mice per group). **e** Working model of microglial-neuronal fractalkine signaling axis. Note that the neuronal-derived CX_3_CL1 either as full length (in case of Non-Tg mice), as complete knockout (in *Cx*_*3*_*cl1*^−/−^ mice), or as the only soluble form with chemokine domain (*Cx*_*3*_*cl1*^105Δ^ mice) differentially regulates the expression of microglial CX_3_CR1 (which is a seven transmembrane G protein-coupled receptor) on the cell surface. This in turn may lead to over-activation of microglia in (*Cx*_*3*_*cl1*^105Δ^ mice) and enhanced neuroinflammation and exacerbation of tau pathology in chemical (LPS) or genetic (hTau) model of tauopathy

Flow cytometric analysis was performed to further explore the possible role of CX_3_CL1^105Δ^ in the regulation of microglial activation in the *Cx*_*3*_*cl1*^105Δ^ mice. Isolated brain myeloid cells were stained with antibodies against CD45 and CD11b to differentiate brain-resident microglia (CD11b^+^CD45^low^) and peripherally derived myeloid cell (CD11b^+^CD45^hi^) population. There were no significant differences in total microglia or peripherally derived myeloid cells within the brains of Non-Tg, *Cx*_*3*_*cl1*^−/−^, or *Cx*_*3*_*cl1*^105Δ^ mice (Fig. 3a, c). However, microglia from the *Cx*_*3*_*cl1*^105Δ^ mice displayed significantly lower cell surface expression for CX_3_CR1 compared to both Non-Tg and *Cx*_*3*_*cl1*^−/−^ mice (Fig. 3b, d). This is despite showing elevated levels of *Cx*_*3*_*cr1* mRNA in the brain (Additional file 1: Figure S1C). Furthermore, the complete lack of fractalkine (in *Cx*_*3*_*cl1*^−/−^ mice) was not sufficient to promote the downregulation of microglial CX_3_CR1 levels (unlike in *Cx*_*3*_*cl1*^105Δ^ mice) (Fig. 3b, d), further supporting previously published reports of receptor downregulation in this model [18].

### *Cx*_*3*_*cl1* deficiency leads to cognitive impairments in aged hTau mice

**Fig. 4:**
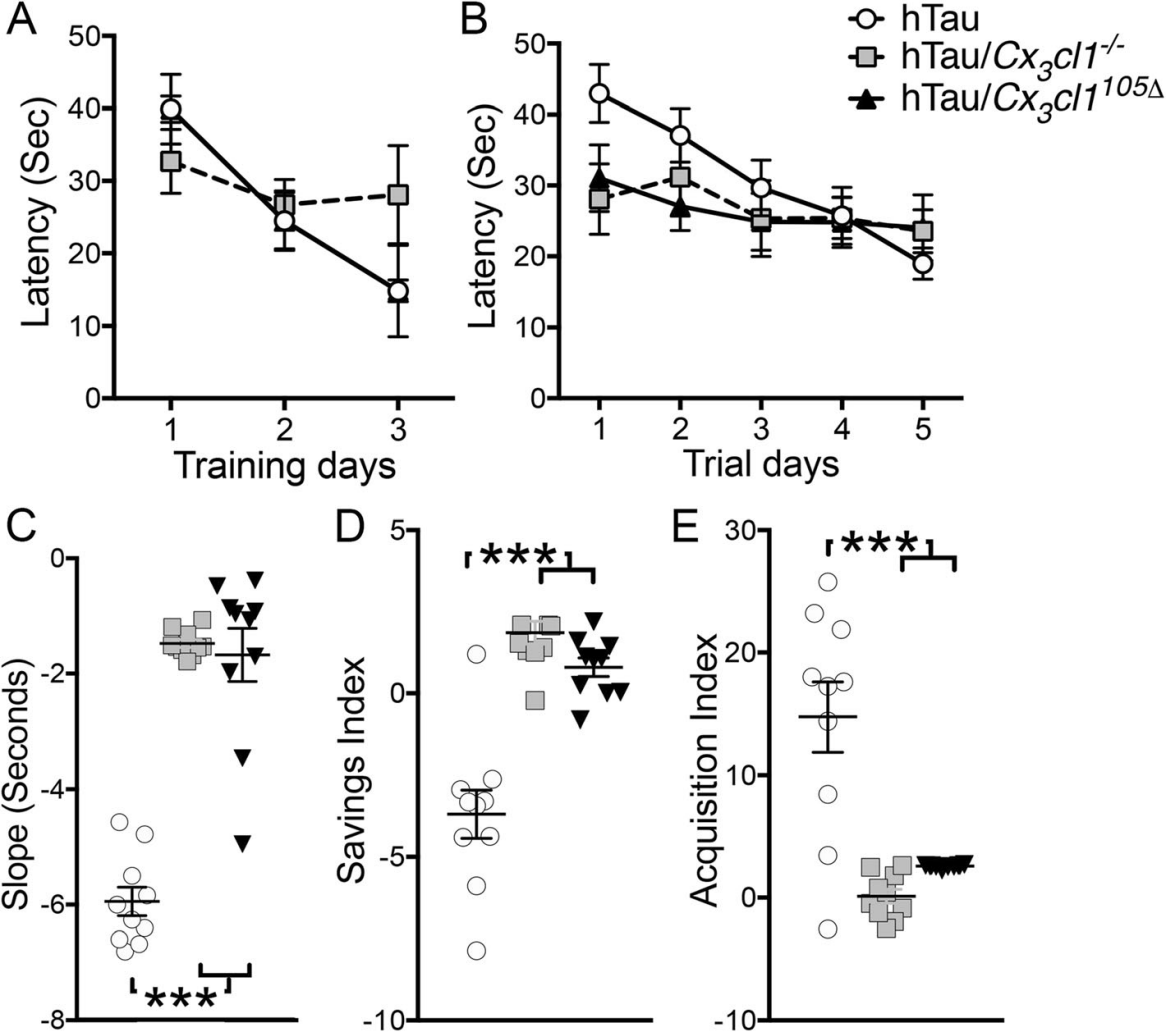
Impaired learning in hTau/*Cx*_*3*_*cl1*^−/−^ and hTau/*Cx*_*3*_*cl1*^105Δ^ mice. Morris water maze was performed on 12-month-old mice. Mice were subjected to a 3-day *visible training paradigm* (**a**), followed by a 5-day hidden trial period (*memory testing*). **b** Where latency to reach the platform was recorded (seconds, sec). **c** Analysis of the linear regression slope adjusted for each genotype revealed that hTau mice learned the task better than hTau/*Cx*_*3*_*cl1*^−/−^ or hTau/*Cx*_*3*_*cl1*^105Δ^ mice over the 5-day hidden trial period. **d** hTau mice had a much lower savings index than hTau/*Cx*_*3*_*cl1*^−/−^ or hTau/*Cx*_*3*_*cl1*^105Δ^ mice during the 5-day hidden trial period. **e** hTau mice show higher acquisition index than hTau/*Cx*_*3*_*cl1*^−/−^ or hTau/*Cx*_*3*_*cl1*^105Δ^ mice during the 5-day testing period. Mean + SEM; one-way ANOVA followed by Tukey’s post hoc test: *p < 0.05, **p < 0.01, ***p < 0.001; *n* = 10 mice per group

Behavioral and cognitive dysfunctions are key clinical deficits in AD and tauopathies. To explore the effect of *Cx*_*3*_*cl1* deficiency in hTau mice, we generated and aged additional cohorts of hTau, hTau/*Cx*_*3*_*cl1*^−/−^, and hTau/*Cx*_*3*_*cl1*^105Δ^ mice to 12 months of age and subjected each group to a Morris water maze behavioral analysis. No statistically significant differences were detected among experimental genotypes with regard to swimming speed or latency to reach the platform during visible or memory trials, respectively (Fig. 4a, b). hTau mice performed significantly better across all five testing days as measured by their respective learning slopes across days 1–5 compared to hTau/*Cx*_*3*_*cl1*^−/−^ or hTau/*Cx*_*3*_*cl1*^105Δ^ mice (Fig. 4c). Detailed analysis of the *acquisition index*, which is a measure comparing learning within each individual trial day across four trials, and the *savings index*, a measure of memory consolidation from the final trial of one testing day to the first trial of the following day, was performed across all 5 days to explore learning trends as previously described [26]. Of interest, hTau mice performed worse day-to-day as measured by savings index, which measures the average memory consolidation from trial 4 on one testing day to trial 1 on the following day, across all 5 days (Fig. 4d). Overall, hTau mice performed better within each respective testing day compared to hTau/*Cx*_*3*_*cl1*^−/−^ or hTau/*Cx*_*3*_*cl1*^105Δ^ mice with regard to new memory formation (acquisition index; Fig. 4e). These data suggest that hTau mice have deficiencies transferring information but attempt to compensate by learning well within each respective testing day, thereby overcoming these impairments and performing better overall than either hTau/*Cx*_*3*_*cl1*^−/−^ or hTau/*Cx*_*3*_*cl1*^105Δ^ mice. Further, the memory impairments induced by *Cx*_*3*_*cl1* deficiency were unable to be overcome by the overexpression of only chemokine domain of CX_3_CL1. We also performed the Y-maze test to assess the working memory and did not find any significant differences in spontaneous alternation ratio between these groups (data not shown).

## Discussion

CX_3_CL1-CX_3_CR1 represents a unique signaling axis between the microglia and neurons, which is profoundly involved in the suppression of innate inflammatory responses. Alterations in fractalkine signaling by chemical or genetic manipulations have dichotomous consequences within the context of canonical AD pathological outcomes. Notably, the absence of fractalkine signaling ameliorates Aβ plaque burden in APPPS1 transgenic mice [30], but exacerbates intraneuronal tau pathology in hTau mouse model of pure tauopathy [17], even though both events likely occur via dysregulation of IL-1β-p38 MAPK signaling pathway [19]. Here, we demonstrate that the expression of the chemokine domain of CX_3_CL1 does not suppress inflammation-induced tau pathology or mitigate microglial responses.

Earlier studies suggested that *Cx*_*3*_*cr1* deficiency increased tau phosphorylation in both LPS and hTau models of tau pathology [17]. This suggests that the presence of CX_3_CR1 may downregulate microglial pro-inflammatory signaling and mitigate inflammation-induced tau hyperphosphorylation. For reasons currently unknown, unlike *Cx*_*3*_*cr1*^−/−^ mice, the *Cx*_*3*_*cl1*^−/−^ mice demonstrate only a modest increase in AT8^+^ tau following LPS administration. Tau phosphorylation in hTau/*Cx*_*3*_*cl1*^−/−^ mice seems to mimic hTau/*Cx*_*3*_*cr1*^−/−^ mice as previously reported [17]. Furthermore, the expression of chemokine domain of fractalkine has virtually no beneficial effects on either LPS-mediated microglial morphological alterations or AT8/AT180 site tau phosphorylation. This observation suggests that in the absence of membrane-bound form, chemokine domain of the fractalkine may, in fact, disrupt normal microglia-neuron signaling, leading to downregulation and/or internalization of fractalkine receptor on the microglial cell surface. Our flow cytometry analysis reveals decreased microglial CX_3_CR1 levels in the *Cx*_*3*_*cl1*^105Δ^ mice compared with Non-Tg or *Cx*_*3*_*cl1*^−/−^ and supports this hypothesis. Interestingly, a previous study observed prolonged downregulation of cell surface CX_3_CR1 on aged microglia in response to LPS [31]. This reduced CX_3_CR1 on the CD11b^+^ microglia corresponded with delayed recovery from sickness behavior, elevated IL-1β induction, and reduced TGFβ [31]. Reduced *Cx*_*3*_*cr1* expression (both mRNA and protein) in monocytes was also reported following septic shock [32]. This loss of monocyte-specific CX_3_CR1, which causes sepsis-induced lethality in humans, compromised this cell’s ability to respond to a fractalkine challenge [32]. While these and our current results suggested pro-inflammatory and pathological effects of reduced CX_3_CR1 expression on the microglial cell surface, the exact intra-microglial alterations in *Cx*_*3*_*cl1*^105Δ^ mice will need to be further explored using isolated microglia and high-throughput single-cell RNA sequencing. Surprisingly, the *Cx*_*3*_*cl1*^105Δ^ mice also had an increased baseline expression of total tau. Finally, there may be the remote possibility of ligand-independent CX_3_CR1 negatively influencing TLR4 signaling in immune cells (in *Cx*_*3*_*cl1*^−/−^ mice) and reducing pro-inflammatory cytokine secretion. Indeed, such un-liganded receptor function was recently reported for progesterone receptor B (without progesterone, acting alone) in the regulation of the function of estrogen receptor-α affecting the proliferation and survival of breast cancer cells following estradiol stimulation [33]. Alternatively, the chemokine domain of CX_3_CL1 has also been shown to induce intracellular signaling independent of CX_3_CR1 via binding to αvβ3 integrins [34].

Similar to our previously reported exacerbation of tau pathology in hTau/*Cx*_*3*_*cr1*^−/−^ mice [17], neuronal fractalkine deletion seems to worsen tau pathology in hTau mice, although differences in tau phosphorylation were only detected at the AT8 (S202) site. Fractalkine deletion elevates the pro-inflammatory response from microglia in hTau/*Cx*_*3*_*cl1*^−/−^ and hTau/*Cx*_*3*_*cl1*^105Δ^ mice compared to hTau mice. Further, cognitive abnormalities are evident in aged hTau/*Cx*_*3*_*cl1*^−/−^ or hTau/*Cx*_*3*_*cl1*^105Δ^ mice regardless of increased production of the soluble chemokine domain of fractalkine in the latter group. Given that hTau mice display impaired performance in the Morris water maze at 12 months of age [35], it is plausible that *Cx*_*3*_*cl1*^105Δ^ overexpression fails to prevent cognitive impairment in hTau mice.

Our results contrast with a previous report where AAV-transduced overexpression of soluble fractalkine rescued several pathological phenomena including the hyperphosphorylation of tau at multiple epitopes and microglial phenotypes in a mouse model of tauopathy, rTg4510 [10]. The discrepancies between our study and Nash et al. could be due to a number of factors including the following: (1) Inducible AAV approach vs. our germline genetic system—their animal model had intact membrane-bound CX_3_CL1, while the *Cx*_*3*_*cl1*^105Δ^ mice did not. (2) Differences in the structure of the soluble fractalkine moiety—in the AAV study, the mucin stalk of fractalkine was included, whereas, in our germline *Cx*_*3*_*cl1*^105Δ^ mice, only the soluble chemokine domain, without the mucin stalk, was present. A previous 3D structural analysis of different domains of CX_3_CL1 has suggested that mucin stalk of CX_3_CL1 is important for the presentation of the chemokine domain to the outer cell membrane and increases adhesive interaction between CX_3_CL1 and CX_3_CR1 [6]. Therefore, lack of the mucin stalk in CX_3_CL^105Δ^ may not be sufficient to restrict LPS-induced or hTau-mediated microglial activation [36]. (3) Presence of endogenous CX_3_CL1 in rTg4510 mice vs. the lack of it in *Cx*_*3*_*cl1*^105Δ^ mice—because of this, the levels of soluble CX_3_CL1^105Δ^ levels in *Cx*_*3*_*cl1*^105Δ^ mice (which is comparable to that of Non-Tg (see Additional file 1: Figure S1B)) may be insufficient compared to significantly higher levels of soluble CX_3_CL1 levels in the AAV study. (4) rTg4510 vs. hTau are two different types of tauopathy mouse models. In rTg4510 only, 4R-Tau with a P301L mutation is expressed and pathological tau is present at 13-fold higher than endogenous levels (AAV study), vs. only an approximate two- to threefold higher expression of all six isoforms, including both 3R and 4R tau, in hTau mice (current study). Based on the data from these two studies, we hypothesize that when there is a robust tau pathology (like in rTg4510 mice), the effect of soluble CX_3_CL1 (containing the mucin stalk) may be beneficial and the benefits are discernable. We also speculate that this beneficial effect could be due, in part, to the contributions from the membrane-bound form of endogenous CX_3_CL1, present in the rTg4510 mice, and the rigidity of the soluble form containing the mucin stalk facilitating the “anti”-inflammation. In contrast, hTau mice do not display as robust tau pathology as rTg4510 mice. Due to the complete lack of membrane-bound CX_3_CL1 in our hTau/*Cx*_*3*_*cl1*^105Δ^ mice, CX_3_CL^105Δ^ may not be as efficient and therefore leads to the downregulation of CX_3_CR1 and exacerbation of neuroinflammation/tau pathology. Together, these interpretations suggest that both membrane-bound CX_3_CL1 and the soluble form of fractalkine may make a concerted effort together to mediate both neuroinflammation and tau pathology.

## Conclusions

Taken together, our data suggest that neuronal expression of only the chemokine domain of fractalkine fails to suppress tau-related pathological outcomes and microglial activation. Our data also suggest that fractalkine acts to tether microglia to neurons and, once this interaction is disrupted, microglia alter their functional phenotype. This signaling benefit is quickly negated under chronic pathological duress and offers little protection from cognitive deficits in advanced stages of the disease. In conclusion, the data presented here suggest that obligatory expression of the chemokine domain of CX_3_CL1 downregulates CX_3_CR1 levels on the microglial cell surface and consequently exacerbates tau pathology. These results could indicate the usefulness of potential therapeutics targeting ADAM10 or ADAM17, which cleave CX_3_CL1, to prevent the formation of excessive soluble CX_3_CL1 as a means to modify disease outcome for tauopathies.

## Additional file

Additional file 1: **Figure S1.** Altered expression of *Cx*_*3*_*cl1* and *Cx*_*3*_*cr1* and protein levels of CX_3_CL1 in non-transgenic, *Cx*_*3*_*cl1*^105Δ^ and. *Cx*_*3*_*cl1*^−/−^ mice. Quantitative real-time PCR analysis showing the expression of *Cx*_*3*_*cl1* and *Cx*_*3*_*cr1* mRNAs (A and C), as well as ELISA analysis for soluble CX_3_CL1 levels in non-transgenic (Non-Tg), *Cx*_*3*_*cl1*^−/−^ and *Cx*_*3*_*cl1*^105Δ^ mice (B). (PDF 371 kb)

## Abbreviations

AAV: Adeno-associated virus
AD: Alzheimer’s disease
APPPS1: Amyloid precursor protein presenilin-1
BAC: Bacterial artificial chromosome
CX_3_CL1: Fractalkine chemokine
Cx_3_cr1: Fractalkine receptor
IHC: Immunohistochemistry
IL-1: Interleukin-1
LPS: Lipopolysaccharide
MAPT: Microtubule-associated protein tau
p38 MAPK: P38 mitogen-activated protein kinase
TGFβ: Transforming growth factor beta
TLR4: Toll-like receptor-4
