# Supplementary material for: Genetically enhancing the expression of chemokine domain of CX3CL1 fails to prevent tau pathology in mouse models of tauopathy
Source: J Neuroinflammation. 2018 Sep 25;15:278. doi: 10.1186/s12974-018-1310-6 (PMC6154806; doi:10.1186/s12974-018-1310-6)
Supplement: Supplementary file 1 — Figure S1. Altered expression of Cx3cl1 and Cx3cr1 and protein levels of CX3CL1 in non-transgenic, Cx3cl1105Δ and. Cx3cl1−/− mice. Quantitative real-time PCR analysis showing the expression of Cx3cl1 and Cx3cr1 mRNAs (A and C), as well as ELISA analysis for soluble CX3CL1 levels in non-transgenic (Non-Tg), Cx3cl1−/− and Cx3cl1105Δ mice (B). (PDF 371 kb) [file 12974_2018_1310_MOESM1_ESM.pdf]

Additional file 1: Figure S1

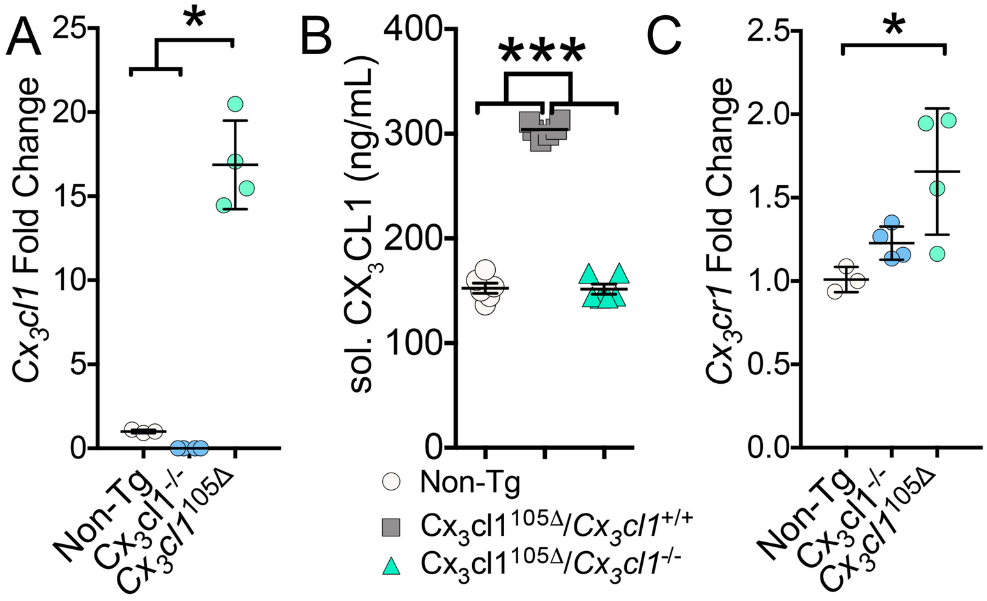

Additional file 1: Figure S1: Altered *Cx₃cl1* and *Cx₃cr1* mRNA expression in non-transgenic, *Cx₃cl1*$^{-/-}$, *Cx₃cl1*$^{105\Delta}$ mice as well as altered soluble CX_3CL1 protein levels in non-transgenic, *Cx₃cl1*$^{105\Delta}$/*Cx₃cl1*$^{+/+}$ and *Cx₃cl1*$^{105\Delta}$/*Cx₃cl1*$^{-/-}$ mice. (A) Quantitative real-time PCR (qRT-PCR) analysis shows significantly elevated *Cx_3cl1* mRNA levels in the brains of *Cx_3cl1*$^{105\Delta}$ mice compared to Non-Tg mice. *Cx_3cl1*$^{-/-}$ serves as negative control with no fractalkine mRNA expression. **(B)** ELISA analysis shows significantly elevated levels of soluble (sol.) fractalkine in the *Cx_3cl1*$^{105\Delta}$/*Cx_3cl1*$^{+/+}$ mice (which expresses $CX_3CL1^{105\Delta}$ along with endogenous CX_3CL1) compared to non-transgenic *Cx_3cl1*$^{105\Delta}$/*Cx_3cl1*$^{-/-}$ (which only expresses $CX_3CL1^{105\Delta}$) mice. **(C)** qRT-PCR analysis shows significantly elevated mRNA levels for *Cx_3cr1* in *Cx_3cl1*$^{105\Delta}$ mice (in *Cx_3cl1*$^{-/-}$ background) compared to Non-Tg, but not *Cx_3cl1*$^{-/-}$ mice. Data displayed as mean $\pm$ SEM; one-way ANOVA followed by Tukey post-hoc test: *$p < 0.05$, ***$p < 0.001$; n=3-6 mice per group).
